# Synaptic basis of rapid antidepressant action

**DOI:** 10.1007/s00406-024-01898-6

**Published:** 2024-09-29

**Authors:** Ege T. Kavalali, Lisa M. Monteggia

**Affiliations:** 1 Department of Pharmacology and the Vanderbilt Brain Institute, Vanderbilt University, 465 21st Avenue South, Suite 7130 Medical Building III, Nashville, TN 37240-7933 USA; 2 Department of Pharmacology and the Vanderbilt Brain Institute, Vanderbilt University, 465 21st Avenue South, Suite 7130 Medical Building III, Nashville, TN 37240-7933 USA

**Keywords:** Ketamine, Synaptic plasticity, Homeostatic plasticity, BDNF, TrkB, Spontaneous neurotransmission, Spontaneous neurotransmitter release

## Abstract

The discovery of ketamine’s rapid antidepressant action has generated intense interest in the field of neuropsychiatry. This discovery demonstrated that to alleviate the symptoms of depression, treatments do not need to elicit substantive alterations in neuronal circuitry or trigger neurogenesis, but rather drive synaptic plasticity mechanisms to compensate for the underlying pathophysiology. The possibility of a rapidly induced antidepressant effect makes therapeutic pursuit of fast-acting neuropsychiatric medications against mood disorders plausible. In the meantime, the accumulating clinical as well as preclinical observations raise critical questions on the nature of the specific synaptic plasticity events that mediate these rapid antidepressant effects. This work has triggered the current growing interest in alternative psychoactive compounds that are thought to have similar properties to ketamine and its action. This review covers our insight into these questions based on the work our group has conducted on this topic in the last decade.

## Introduction

**Fig. 1:**
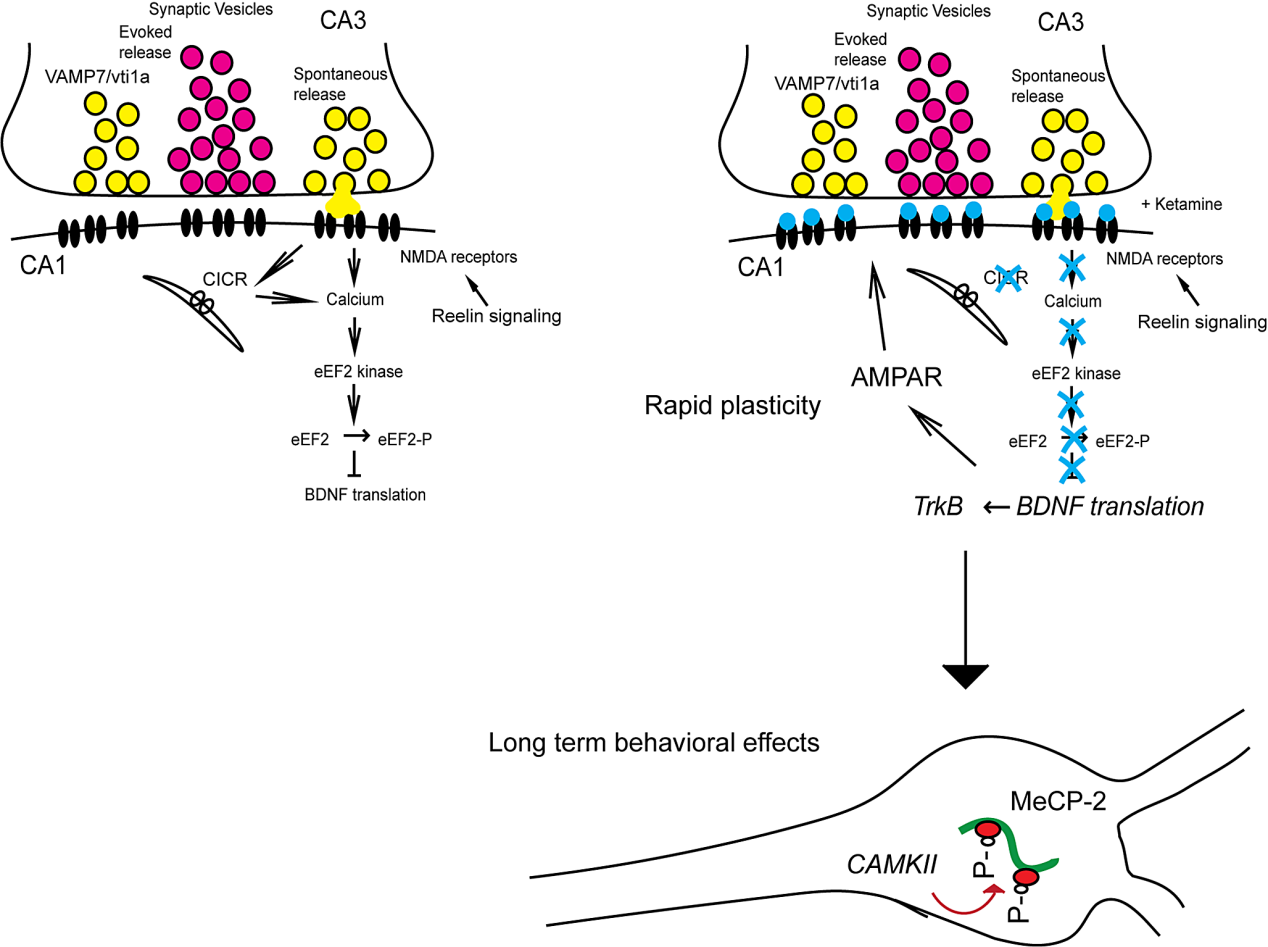
The model depicts key synaptic signal transduction cascades uncovered by our work that drive ketamine’s rapid antidepressant action. In this model, ketamine mediated suppression of N-methyl-D-aspartate (NMDA) receptor-mediated spontaneous neurotransmission leads to rapid augmentation of dendritic protein translation via decrease in eEF2 kinase activity and eEF2 phosphorylation. Spontaneous release driven by non-canonical SNARE molecules vti1a and VAMP7 appears to be critical for the form of glutamate release that elicits this resting NMDAR signaling. The maintenance of NMDA receptor mediated transmission by Reelin signaling is essential as this sustains baseline NMDA receptor mediated transmission and renders it susceptible to open channel block by ketamine. Ca^2+^-induced Ca^2+^ release (CICR) also plays an important role in amplifying Ca^2+^ signals originating from the resting level of NMDAR activity. Amongst the rapidly synthesized and secreted proteins is brain-derived neurotrophic factor (BDNF), which signals via its canonical receptor tropomyosin receptor kinase B (TrkB), leading to potentiation of α-amino-3-hydroxy-5-methyl-4-isoxazole propionic acid (AMPA) receptor mediated glutamatergic transmission to drive the rapid antidepressant effects. Downstream of BDNF signaling, phosphorylation of transcriptional regulator MeCP2 and subsequent transcriptional events are required to prolong the rapid antidepressant effects for weeks beyond their initial phase

Clinical observations regarding ketamine’s rapid antidepressant action build a clear framework regarding potential mechanisms that underlie this effect. A brief infusion leads to rapid antidepressant effects that begin within an hour and are sustained for a week or longer following ketamine’s removal [1, 2]. These unequivocal clinical observations vouch for a tractable mechanistic model that is amenable to systematic investigation. In our studies, we aimed to reverse-engineer ketamine action to build a causal sequence of molecular events that not only account for the rapid effects that emerge within hours but also its longer lasting effects that are maintained over weeks. Our studies have focused on examining synaptic targets that drive ketamine’s antidepressant action with the aim of uncovering the mechanistic basis of clinical observations [3–6] (Fig. 1). This work has uncovered a key role for N-methyl-D-aspartate (NMDA) receptor mediated spontaneous neurotransmission, its maintenance by Reelin signaling as well as rapid augmentation of dendritic protein translation following suppression of this spontaneous glutamate release-driven “resting” NMDA receptor activity by ketamine. Amongst the rapidly synthesized and secreted proteins is brain-derived neurotrophic factor (BDNF), which signals via its canonical receptor tropomyosin receptor kinase B (TrkB), leading to potentiation of α-amino-3-hydroxy-5-methyl-4-isoxazole propionic acid (AMPA) receptor mediated glutamatergic transmission to drive the rapid antidepressant effects. Here, the role of BDNF signaling emerged as a point of potential convergence with classical antidepressants. Importantly, our studies demonstrated that the synaptic plasticity elicited by ketamine was a form of homeostatic plasticity akin to those triggered by chronic manipulations of activity rather than a form of Hebbian plasticity such as Long Terms Potentiation (LTP) that is synapse specific. This mechanistic model also explains the inefficacy of memantine as an antidepressant that overall shares similar properties as ketamine. We found that memantine, unlike ketamine, is not efficacious in suppressing NMDA receptor activity at near resting membrane potentials, highlighting the key role played by this spontaneous glutamate release-driven “resting” NMDA receptor signaling in the process. We can also link this initial sequence of events to longer effects, which require transcriptional regulation of MeCP2 phosphorylation elicited downstream of BDNF signaling (Fig. 1). In this review, we will provide a systematic survey of experiments that built this mechanistic model for ketamine action.

## Synaptic mechanisms underlying ketamine action

In initial studies, we addressed whether ketamine’s rapid antidepressant action could be recapitulated in preclinical animal models and, if so, whether the behavioral response required neurotrophic factor BDNF signaling, as in previous work with classical slow acting antidepressants such as selective serotonin reuptake inhibitors (SSRIs) [7]. Here, we were able to recapitulate the rapid behavioral effect with ketamine as well as other NMDA receptor blockers MK-801 and CPP. We also observed a robust increase in BDNF levels and a behavioral response that requires BDNF (as brain specific BDNF conditional knock out mice did not respond). These findings raised the question as to how ketamine, a blocker of NMDA receptors, could raise BDNF levels, and if this rise in BDNF triggered some form of synaptic plasticity. In a series of experiments, we observed that these fast-acting antidepressant effects depended on the rapid synthesis of BDNF. We found that the ketamine-mediated blockade of NMDAR at rest deactivates eukaryotic elongation factor 2 (eEF2) kinase (also called CaMKIII), resulting in reduced eEF2 phosphorylation and de-suppression of protein translation, including that of BDNF. These initial findings put forward the hypothesis that the regulation of protein synthesis by spontaneous neurotransmission may serve as a viable therapeutic target for the development of fast-acting antidepressants.

In a subsequent study, we explored a potential link between ketamine application and synaptic plasticity [8]. At the time, the impact of spontaneous neurotransmission on neuronal plasticity remained poorly understood. Here, we were able to show that acute suppression of spontaneous NMDA receptor-mediated neurotransmission potentiates synaptic responses at CA3-CA1 synapses of rat and mouse hippocampus. This potentiation required protein synthesis, BDNF expression, eEF2 kinase function, and increased surface expression of AMPA receptors. Our behavioral studies using genetic knockouts of BDNF, TrkB, as well as eEF2 kinase linked this same synaptic signaling pathway to the fast-acting antidepressant responses elicited by ketamine. These findings uncovered an unexpectedly dynamic impact of spontaneous glutamate release on synaptic efficacy and provided insight into a key synaptic substrate for rapid antidepressant action.

We further followed these initial observations and investigated the ketamine mediated antidepressant response and the resulting synaptic potentiation in juvenile animals [9]. We observed that ketamine did not produce an antidepressant response in juvenile animals in the novelty suppressed feeding or the forced swim test. In addition, ketamine application failed to trigger synaptic potentiation in hippocampal slices obtained from juvenile animals, unlike its action in slices from adult animals. The inability of ketamine to trigger an antidepressant response or subsequent synaptic plasticity processes suggested a developmental component to ketamine mediated antidepressant efficacy. We could also show that the NMDAR antagonist AP5 triggers synaptic potentiation in mature hippocampus replicating the action of ketamine, demonstrating that global competitive blockade of NMDARs is sufficient to trigger this effect. Taken together, these findings suggested that global blockade of NMDARs in developmentally mature hippocampal synapses is required for the antidepressant efficacy of ketamine.

In addition to these findings, two lines of experiments also helped bolster the premise that ketamine induced synaptic plasticity and the ensuing antidepressant effects indeed depends on NMDA receptor block. First, we reassessed the claim that the ketamine metabolite (2R,6R)-hydroxynorketamine (HNK) is essential for the antidepressant effects of ketamine in mice and acts in an NMDAR-independent manner [10]. This study did not specify an alternative mechanism but replicated our observation that ketamine application resulted in potentiation of AMPAR mediated synaptic responses. In our investigation, we found that (2R,6R)-HNK blocks synaptic NMDARs in a similar manner to its parent compound, and we showed that the effects of (2R,6R)-HNK on intracellular signaling are coupled to NMDAR inhibition. Overall, these data demonstrated that (2R,6R)-HNK inhibits synaptic NMDARs and subsequently elicits the same signal transduction pathway previously associated with NMDAR inhibition by ketamine [11]. These findings suggest that ketamine produces rapid antidepressant action by blocking NMDARs activated by spontaneous glutamate release and triggering the intracellular signaling that leads to the synaptic potentiation at CA3-CA1 synapses. As ketamine is metabolized, (2R,6R)-HNK then continues to block resting NMDAR activity, extending the drug action and providing an explanation as to why ketamine has antidepressant effects for several days that are not seen with other NMDAR blockers.

Second, the requirement for the NMDA receptor was further supported by our more recent study demonstrating that the synaptic signaling mediated by the secreted glycoprotein Reelin is required for ketamine’s rapid antidepressant action [12]. Reelin acts as a neuromodulator in the adult brain by regulating pre- and postsynaptic machinery via its receptors, apolipoprotein E receptor 2 (Apoer2) and very-low-density lipoprotein receptor (VLDLR) and increases tyrosine phosphorylation in Disabled-1 (DAB1). The Reelin pathway regulates pre- or postsynaptic function through its downstream signaling pathways in the adult brain. In presynaptic terminals, the Reelin-Apoer2 pathway activates phosphoinositide 3-kinase (PI3K) and increases Ca^2+^ release from intracellular stores, which in turn mobilizes VAMP7-containing synaptic vesicles and augments spontaneous release [13]. At the postsynaptic sites, Reelin’s binding to Apoer2 reciprocally activates DAB1 and Src family kinases (SFKs). Subsequently, the activated SFKs increase tyrosine phosphorylation of NMDAR subunits, GluN2A and GluN2B, and increase NMDAR open probability [14]. To test this premise, we used mouse models with genetic deletion of Reelin or apolipoprotein E receptor 2 (Apoer2), as well as pharmacological inhibition of their downstream effectors, Src family kinases (SFKs) or phosphoinositide 3-kinase. In our investigation, we found that the disruption of Reelin-mediated synaptic signaling alters ketamine-triggered synaptic plasticity and behavioral effects. We also found that disruption of Reelin, Apoer2, or SFKs blocks ketamine-driven behavioral changes and synaptic plasticity in the hippocampal CA1 region. These results suggested that maintenance of baseline NMDA receptor function by Reelin signaling has a key permissive role required for ketamine’s antidepressant effects supporting the requirement of NMDA receptor signaling [12].

### BDNF-TrkB signaling dependent synaptic plasticity is essential for ketamine action

As described above, our studies were the first to identify BDNF and its receptor TrkB as necessary for the antidepressant effects and underlying ketamine-induced synaptic potentiation in the hippocampus [7, 8]. However, these earlier studies did not address whether a specific synaptic locus, if any, is required to elicit the antidepressant action. To answer this question, we deleted BDNF or TrkB in presynaptic CA3 or postsynaptic CA1 regions of the Schaffer collateral pathway and investigated the rapid antidepressant action of ketamine. The deletion of BDNF in CA3 or CA1 blocked the ketamine-induced synaptic potentiation. In contrast, ablation of TrkB only in postsynaptic CA1 eliminated the ketamine-induced synaptic potentiation. We were also able to demonstrate that BDNF-TrkB signaling in CA1 is required for ketamine’s rapid behavioral action [15]. Moreover, ketamine application elicited TrkB activation, coupled with dynamin-1 mediated endocytosis of this receptor, that was critical in downstream signaling to leading to rapid synaptic effects. Taken together, these data demonstrated a requirement for BDNF-TrkB signaling in CA1 neurons in ketamine-induced synaptic potentiation and identified a specific synaptic locus in eliciting ketamine’s rapid antidepressant effects. The requirement for BDNF-TrkB signaling in the CA1 region highlights the role of hippocampus in mediating the antidepressant effect but this result does not exclude the involvement of other regions as well [15].

Here, it is important to note that while BDNF signaling can modulate Hebbian types of plasticity such as LTP [16, 17], the study described above showed that BDNF signaling is absolutely essential for ketamine induced plasticity. This finding, taken together with earlier work on the manipulation of spontaneous neurotransmission [18], and more recent work directly examining the intersection of LTP and ketamine-mediated plasticity, strongly indicate that the two forms of plasticity operate independently and do not overlap with each other [19]. This conclusion is critical as it indicates that ketamine enhances glutamatergic transmission through homeostatic plasticity without disrupting Hebbian plasticity mechanisms and maintains synaptic integrity and LTP even under stress conditions, supporting its use in treating depression with minimal impairments in learning and memory processes.

### Insights into NMDA receptor mediated signaling at rest

The studies described so far raise the question whether NMDA receptors — normally thought to be silenced at near resting membrane potentials — can signal in response to quantal spontaneous release. Prior to our work on ketamine, we had investigated whether NMDA receptor activation at near resting membrane potentials could maintain detectable synaptic currents [20]. This was a critical open question as it is well-established that under physiological conditions NMDA receptor activation requires coincidence of presynaptic glutamate release and postsynaptic depolarization due to the voltage-dependent block of these receptors by extracellular Mg^2+^. Therefore, spontaneous neurotransmission, in the absence of action potential firing, was not expected to lead to significant NMDA receptor activation [20]. However, this expectation contradicted the observation that blockade of NMDARs in the absence of action potentials could trigger homeostatic plasticity. In subsequent experiments, we were able to detect a NMDA current component comprising approximately 20% of the charge transfer during an average miniature excitatory postsynaptic current (mEPSC) detected at resting membrane potentials. In addition, we also provided evidence that potential AMPA receptor-driven local depolarizations were not necessary to drive NMDA receptor activity at rest suggesting that NMDA receptors significantly contribute to signaling near resting membrane potentials in the absence of dendritic depolarizations or concomitant AMPA receptor activity driven solely by spontaneous glutamate release [20].

The finding that NMDA receptors could indeed maintain electrical neurotransmission at near resting membrane potentials provided the motivation for the subsequent study to examine whether ketamine was effective in blocking these resting NMDA receptor mediated currents and whether there were any differences between the actions of ketamine and memantine [21]. This is a key question as memantine has been shown not to impart any rapid antidepressant effects in the clinic, despite having largely similar properties of block of open NMDA receptors similar to ketamine. Here, we recapitulated the ketamine and memantine clinical findings in mice, showing that ketamine, but not memantine, has antidepressant-like effects in behavioral models [21]. Using electrophysiology to study hippocampal neurons, we showed that ketamine and memantine effectively block NMDAR-mediated miniature excitatory postsynaptic currents in the absence of Mg^2+^. However, in physiological levels of extracellular Mg^2+^, we identified key functional differences between ketamine and memantine in their ability to block NMDAR function at rest [21]. This differential effect of ketamine and memantine extends to intracellular signaling coupled to NMDAR at rest, in that memantine does not inhibit the phosphorylation of eEF2 or augment subsequent expression of BDNF, which are critical determinants of ketamine-mediated antidepressant efficacy. These results demonstrated significant differences between the efficacies of ketamine and memantine on NMDAR-mediated neurotransmission that impact downstream intracellular signaling, which we hypothesize is the trigger for rapid antidepressant responses. These data provide a framework on the functional requirements of NMDAR-mediated neurotransmission as a critical determinant necessary to elicit rapid antidepressant responses [21]. These findings also provide a counterpoint to proposals that ketamine action involves ketamine mediated block of NMDARs on inhibitory interneurons. Block of these inhibitory interneuron resident NMDARs is postulated to suppress tonic release of GABA and disinhibit activity of the target excitatory neurons [22]. However, it is important to note that disinhibitory effects similar to ketamine action can also be elicited by memantine [23] although memantine does not share ketamine’s efficacy as a fast-acting antidepressant.

### Properties of homeostatic synaptic plasticity triggered by ketamine

Subsequent studies on resting NMDA receptor signaling have focused on postsynaptic as well as presynaptic aspects of spontaneous release and contributed to our understanding of how spontaneous glutamate release driven NMDA receptor activity can elicit biochemical signaling and synaptic plasticity. First, to examine the properties of Ca^2+^ signals using hippocampal neurons labeled with the fluorescent Ca^2+^ probes, we visualized action potential-independent transients in dendritic regions adjacent to fluorescently labeled presynaptic boutons in physiological levels of Mg^2+^ [24]. These Ca^2+^ transients required NMDA receptor activity driven by spontaneous glutamate release. Importantly, inhibition of calcium-induced calcium release (CICR) suppressed these transients and elicited synaptic scaling, a process which required protein translation and eEF2 kinase activity. These results supported a critical role for CICR in amplifying NMDA receptor-driven Ca^2+^ signals that occur independent of activity. These findings also demonstrated that this resting Ca^2+^ signaling is essential for the maintenance of synaptic homeostasis.

While this work implicated spontaneous neurotransmission in homeostatic synaptic scaling, few studies have selectively manipulated spontaneous neurotransmission without substantial changes in evoked neurotransmission to study this function in detail. Therefore, next, we manipulated spontaneous glutamate release by targeting the non-canonical synaptic vesicle SNAREs Vps10p-tail-interactor-1a (vti1a) and vesicle-associated membrane protein 7 (VAMP7) to specifically inhibit spontaneous release events and probe whether these events signal independently of evoked release to the postsynaptic neuron. We found that loss of vti1a and VAMP7 impairs spontaneous high-frequency glutamate release and augments unitary event amplitudes by reducing postsynaptic eEF2 kinase activity after the reduction in NMDAR activity. Presynaptic, but not postsynaptic, loss of vti1a and VAMP7 occluded NMDAR antagonist-induced synaptic potentiation in an intact circuit, confirming the role of these vesicular SNAREs in setting synaptic strength. Together, these results demonstrated that spontaneous neurotransmission signals independently of stimulus-evoked release and highlight its role as a key regulator of postsynaptic efficacy [18]. This premise was further cemented by the using a quadruple knockdown strategy to reduce levels of proteins of the all four members of the soluble calcium-binding double C2 domain (Doc2)-like protein family to selectively reduce spontaneous neurotransmission. In this system, activity-evoked responses appear normal while both excitatory and inhibitory spontaneous events exhibit reduced frequency. Excitatory miniature postsynaptic currents (mEPSCs), but not miniature inhibitory postsynaptic currents (mIPSCs), increase in amplitude after quadruple knockdown. This increase in synaptic efficacy correlates with reduced phosphorylation levels of eEF2 and requires the presence of eEF2 kinase. Together, these data suggest that spontaneous neurotransmission independently contributes to the regulation of synaptic efficacy, and action potential-evoked and spontaneous neurotransmission can be segregated at least partially on a molecular level [25].

Findings outlined above highlight the key role played by eEF2 kinase signaling and its regulation by spontaneous glutamate release for the rapid antidepressant action of ketamine. To further elucidate the mechanisms that link eEF2K function to plasticity, we used genetic, electrophysiological, and pharmacological strategies to investigate the role of eEF2K in synaptic function and find that acute, but not chronic, inhibition of eEF2K activity induces rapid synaptic scaling in the hippocampus. Retinoic acid (RA) signaling also elicits a similar form of rapid synaptic scaling in the hippocampus [26], which we observe is independent of eEF2K function. The RA signaling pathway is not required for ketamine-mediated antidepressant action; however, direct activation of the retinoic acid receptor α (RARα) evokes rapid antidepressant action resembling ketamine. Overall, in this work our findings showed that ketamine and RARα activation independently elicit a similar form of multiplicative synaptic scaling that is causal for rapid antidepressant action [27].

### Mechanisms underlying sustained effects of ketamine

The rapidly acting antidepressants ketamine and scopolamine exert behavioral effects that can last from several days to more than a week in some patients [28]. The molecular mechanisms underlying the maintenance of these antidepressant effects remain poorly understood. In a recent study, our group showed that phosphorylation of the transcriptional regulator methyl-CpG-binding protein 2 (MeCP2) at Ser421 (pMeCP2) is essential for the sustained, but not the rapid, antidepressant effects of ketamine and scopolamine in mice. Our results reveal that pMeCP2 is downstream of BDNF, a critical factor in ketamine and scopolamine antidepressant action. In addition, we showed that pMeCP2 is required for the long-term regulation of synaptic strength after ketamine or scopolamine administration. These results indicated that pMeCP2 and associated synaptic plasticity are essential determinants of sustained antidepressant effects [29]. Importantly, in these experiments revealed that ketamine administration produces a priming effect (dependent of MeCP2 phosphorylation) where subsequent ketamine treatment causes significantly augmented plasticity implying that ketamine imparts a form of metaplasticity [29]. This result provided insight into the earlier clinical findings that repeated ketamine infusions are more effective than a single infusion in patients with major depressive disorder.

## Insight into principles of rapid antidepressant action

In this article, we provided an overview of our studies into the mechanisms of rapid antidepressant action mediated by ketamine. Our work systematically focused on synaptic targets that elicit ketamine induced plasticity and how it affects behavior. We used high resolution electrophysiology and optical imaging as well as long duration field recordings to probe the mechanisms of plasticity taking advantage of various reverse genetic manipulations of key signaling factors. Despite the extensive work our group and others have conducted over the past decade, several key questions remain. Does treatment with ketamine or other potential rapidly acting antidepressants directly counter the underlying pathology of depression and thus reverse the symptoms? Do the mechanisms underlying major depressive disorder overlap with synaptic mechanisms targeted by ketamine? Our answer is equivocal. We cannot exclude a role for NMDA receptor mediated signaling targeted by ketamine in the pathophysiology of depression. However, we can show that rapid induction of depressive symptoms by administration of the acetylcholinesterase inhibitor physostigmine, can be reversed by ketamine via non-overlapping synaptic mechanisms [30]. This recent result strongly indicates that the action of ketamine, or similar therapeutics, do not necessarily need to specifically counter the disease pathology to alleviate symptoms and foster treatment advance.

Another aspect of ketamine action is the role of distinct brain regions in antidepressant effects. Our studies have primarily focused on the hippocampus as specific impairment of BDNF-TrkB signaling and associated synaptic plasticity within the hippocampus is sufficient to impair the antidepressant effect. However, this result does not negate the potential role of other regions such as prefrontal cortex or lateral habenula in conjunction with the hippocampus in this response. For instance, it is postulated that ketamine may directly correct aberrant activity within the lateral habenula and counter underlying pathophysiology to elicit antidepressant effects [31, 32]. It is critical to note that depression is not a monolithic disorder and has a diverse array of causes. In this regard, it is difficult to reconcile ketamine’s efficacy against multiple forms of depression with its ability to correct aberrant activity within a single brain region [33].

Furthermore, mechanisms underlying long term efficacy of ketamine remain unclear. While our studies show a transcriptional mechanism mediated by MeCP2 phosphorylation downstream of BDNF signaling as a major trigger for effects that exceed the initial rapid response [29], the exact mechanisms that may be required to prolong this effect are unknown. For instance, why do the antidepressant effects subside within a few weeks at most? Is this a passive process or an active re-balancing of signaling mechanisms? Addressing these questions will help design approaches to maintain therapeutic effects for longer periods without repeated administration.

Finally, do psychedelics and related compounds also tap into similar synaptic mechanisms to elicit their rapid effects? Are the same synaptic plasticity or metaplasticity paradigms applicable for the potentially beneficial effects of these compounds against mood disorders? The initial answers to these questions appear to be affirmative and suggest a role for a form of synaptic metaplasticity [34], although this area of research needs further mechanistic insight [33]. We hope our past and current studies will provide a roadmap to decipher mechanisms underlying the beneficial effects of these therapies, elucidate the pathophysiology of these devastating mood disorders, and help design of novel rapidly acting, effective and sustainable strategies for their treatment.

## Data Availability

This is a review article, data availability is not applicable as no data was generated for this work.

## Appendix

**Spontaneous neurotransmission:** Neurotransmitter release and subsequent signaling that occur independent of neuronal activity, especially in the absence of presynaptic action potentials.

**Vtia1a:** Vps10p-tail-interactor-1a (vti1a) is a mammalian homolog of the yeast SNARE (Soluble N-ethylmaleimide-Sensitive Factor Attachment Receptor) protein vti1p, which is involved in transport between the endosome and the trans-Golgi network. In neurons, vti1a is localized to cell bodies as well as presynaptic terminals, and a splice variant of this protein is enriched in synaptic vesicles. Vti1a preferentially traffics under resting conditions during spontaneous neurotransmission.

**VAMP7:** Vesicle Associated Membrane Protein 7, also known as tetanus toxin-insensitive VAMP (TI-VAMP), is a vesicle associated SNARE protein. It is predominantly present in the Golgi apparatus, endosomes and lysosomes. In developing neurons, VAMP7 is localized to growth cones and regulates neurite outgrowth. VAMP7 is expressed throughout the adult brain, typically in somatodendritic compartments, but is found in presynaptic terminals, responsible for regulating release in response to elevated levels of resting Ca^2+^.

**BDNF:** Brain-derived neurotrophic factor (BDNF) is a neuropeptide that plays numerous important roles in neuronal development and plasticity. In the last two decades, BDNF has emerged as a key factor that underlies the efficacy of neuropsychiatric treatments in addition to being a critical factor for synaptic development and plasticity.

**eEF2:** Eukaryotic elongation factor 2 promotes translocation of the ribosome during protein translation. This protein is inactivated by phosphorylation by its dedicated eEF2 kinase.

**Quantal Release:** In contemporary neurophysiology this term typically refers to neurotransmitter release carried out by fusion of individual synaptic vesicles.

**Synaptic Scaling:** A form of synaptic plasticity where strengths of individual synaptic inputs into a neuron are scaled-up or -down via multiplication with a single factor. In this way, synaptic scaling-mediated plasticity preserves relative strengths of synapses across a neuron.
